# Association of waiting and consultation time with patient satisfaction: secondary-data analysis of a national survey in Peruvian ambulatory care facilities

**DOI:** 10.1186/s12913-019-4288-6

**Published:** 2019-07-01

**Authors:** Christoper A. Alarcon-Ruiz, Paula Heredia, Alvaro Taype-Rondan

**Affiliations:** 1 Unidad de Investigación para la Generación y Síntesis de Evidencias en Salud, Universidad San Ignacio de Loyola, Lima, Peru; 2 Faculty of Medicine, Universidad Ricardo Palma, Lima, Peru

**Keywords:** Patient satisfaction, Ambulatory care, Survey research, Consultation, Hispanic health

## Abstract

**Background:**

Research suggested that waiting time and consultation time are associated with overall patient satisfaction concerning health services. However, there is a lack of information regarding this subject in Latin American countries, where particular aspects of health systems and population characteristics could modify this association. Our aim was to evaluate the association of waiting time and consultation time with patient satisfaction, in Peruvian ambulatory care facilities and propose a cut-off points of waiting and consultation time based on patient satisfaction.

**Methods:**

Cross-sectional secondary data analysis of the National Survey on User Satisfaction of Health Services (ENSUSALUD-2015), a national-wide survey with a probabilistic sample of 181 Peruvian ambulatory care facilities. Patient satisfaction, waiting time, consultation time, and sociodemographic variables were collected from the ENSUSALUD-2015. All variables were collected by survey directly to patients, from the selected ambulatory care facilities, after their consultation. Complex survey sampling was considered for data analysis. In the association analysis, we grouped the waiting time and consultation time variables, every 10 min, because for it is more relevant and helpful in the statistical and practical interpretation of the results, instead of the every-minute unit.

**Results:**

The survey was performed in 13,360 participants. Response rate were 99.8 to 100% in the main variables. Waiting time (for every 10 min) was inversely associated with patient satisfaction (aOR: 0.98, 95% CI: 0.97–0.99), although the aOR was lower among those who reported a waiting time ≤ 90 min (aOR: 0.92, 95% CI: 0.89–0.96). Consultation time (for every 10 min) was directly associated with patient satisfaction (aOR: 1.59, 95% CI: 1.26–2.01), although the aOR was higher among those who reported a consultation time ≤ 15 min (aOR: 2.31, 95% CI: 1.66–3.21).

**Conclusion:**

In Peruvian ambulatory care facilities, both waiting time and consultation time showed an association with overall patient satisfaction, which was stronger in the first 90 min of waiting time and in the first 15 min of consultation time. This should be taken into consideration when designing interventions to improve waiting times and consultation times in ambulatory care facilities from Peru or from similar contexts.

## Background

Overall patient satisfaction with health services (Hereinafter referred to as patient satisfaction) is a critical component in the evaluation of health care services [1, 2]. Furthermore, greater patient satisfaction is associated with better patient health outcomes such as higher rates of fulfilling physician’s instructions [3] and lower mortality rate [4, 5]. However, there is a need for assessing high-quality interventions to improve patient satisfaction [6], and describe the patient’s perceptions of their experiences and their satisfaction [7].

Waiting and consultation times in ambulatory care facilities (ACFs) are associated with patient satisfaction [8–10], so that longer waiting times and shorter consultation times could cause impaired access to healthcare [11] and a decrease of patient’s willingness to return to the ACF, which ultimately has an impact on continuity of care [12]. Both variables are of great interest because they are relatively easy to modify by interventions focused on the attendance process [13]. Thus, their modification has the potential to improve patient satisfaction at a low cost. Nevertheless, the “ideal” waiting and consultation time is not yet well defined. So, it is important to determine a cut-off points of both times where the patients’ overall satisfaction significantly can be modified.

Previous studies which have evaluated the association of waiting time and consultation time with patient satisfaction were performed mostly in high-income countries [10, 14–17]. However, middle and low-income Latin American countries use to have long waiting times, possibly due to their crowded health system [18–20]. Additionally, these associations could show different patterns, due to particular aspects of the health systems of those countries [21]. Moreover, patients in Latin American counties could want to invest a short amount of time in their healthcare, in order to prevent work problems, since employment in these countries commonly have non-flexible work shifts, low-tolerance for absenteeism, and labor instability [22, 23].

Thus, this study aimed to evaluate the association of waiting time and consultation time with patient satisfaction, in Peruvian ACFs and propose a cut-off points of waiting and consultation time based on patient satisfaction.

## Methods

### Study design

We performed a cross-sectional secondary data analysis of a public dataset from the National Survey on User Satisfaction of Health Services 2015 (ENSUSALUD-2015). It was recollected by the National Superintendence of Health (SUSALUD, in Spanish) and the National Institute of Statistics and Informatics (INEI, in Spanish). ENSUSALUD-2015 is a national-wide survey carried out in Peruvian ACFs in 2015, which aims to assess the ACFs’ users’ perception about the care provided.

### Context

ACFs are health facilities which administer health services to individuals who do not require hospitalization into a health care facility. In Peru, ACFs belong to any of four health systems: 1) Ministry of Health and Regional Governments (MOH-RG), which provide health care mainly to low-income people. It is supported by the Peruvian Government, and it works in three different levels: Local, regional and national; 2) Social Security (EsSalud), financed by the Ministry of Labor, which provides health care to formal workers, former workers, and their relatives. It operates using its own centers of health; 3) Armed Forces (Marines, army and aviation) and Police, financed by the Ministry of Defense and the Ministry of Interior, respectively. All of them works independently from each other. They provide health care to armed forces and police members, and their relatives; 4) The private sector, financed by users with private insurance or by paying at the same moment of attendance [21].

ACFs are divided into three different levels: Level I ACFs provide basic health care and takes care of the most frequent low complexity problems. Level II ACFs have the capacity to resolve some surgical problems and the ones transferred from level I ACFs. Level III ACFs are reserved for the attendance of complex conditions requiring specialized medical procedures [24]. In most cases, with the exception of the private sector, patients should be referred from lower levels in order to assist to higher levels ACFs. The staffing in the ACFs depends on the level of ACFs, but in all cases, the patient is attended by a medical doctor.

### Participants

The ENSUSALUD-2015 surveyed a representative sample of users after their medical consultation at Peruvian ACFs. A probabilistic sampling across all 25 Peruvian regions was conducted. This sampling was performed in a two-stage process: First, a random selection of Peruvian ACFs (from level I, II, and III) in each health system was performed. Then, outpatient users of each ACFs were selected systematically, considering the number of daily attendances in each ACF. The survey was available to users with age ≥ 15 years old, who had a medical consultation in the ACF, and accepted to be surveyed [25].

### Procedures

Surveys were completed between May and July 2015. A trained pollster surveyed each user immediately following their medical consultation. The pollsters introduced themselves to users and asked them for their consent to conduct the survey. Then, pollsters read each question and their alternatives to the user. No incentive was offered to participants to respond to the survey. The database and details of the survey are public on the INEI webpage (http://iinei.inei.gob.pe/microdatos/).

### Variables

#### Outcome: patient satisfaction

The outcome for this study was patient satisfaction. It was evaluated using the question: “Regarding the health care service received today in this health facility, how would you rate your satisfaction level?” (“*Respecto al servicio recibido el día de hoy en este establecimiento, ¿Cómo calificaría usted su nivel de satisfacción?*”, in Spanish). It had the following response options: Very satisfied, satisfied, neither satisfied nor dissatisfied, dissatisfied, and very dissatisfied. All questions in the survey were asked in the patient’ maternal language.

For the purpose of the analysis and because of skewed data distributions, this item was dichotomized into two categories: “Satisfied” (if the participant answered “very satisfied” or “satisfied”) or “Not satisfied” (if the participant answered “neither satisfied nor dissatisfied”, “dissatisfied”, or “very unsatisfied”). Previous studies have used this methodology [5, 26].

#### Exposures: waiting time and consultation time

The exposures of this study were waiting time and consultation time. Both were analyzed as continuous variables. To evaluate waiting time, we considered the following questions: “At what time did you arrive at the health facility?” And “At what time did you enter the physician’s office?” Both variables were recorded in minutes. The difference between these two times (time of entry to the physician’s office minus the time of arrival to the health facility) was defined as waiting time.

Consultation time was measured with the question: “How long was the time, from the moment you have admitted the physician’s office to the time you left the physician’s office?” This variable was recorded in minutes.

### Other variables

Sociodemographic variables included in the analysis were: age (in years), sex (male or female), having finished secondary education (yes or no), wealth (measured with the question: “How much is, approximately, the monthly family income?” Later, this numeric variable was categorized in its 1st/2nd quintile [with the lower income, between 80 and 1500 PEN] and 3rd/4th/5th quintile [with the higher income, between 1501 and 50,000 PEN]) (1 PEN = 0.30 USD, approximately), having a chronic illness (evaluated with the yes/no question: “Do you suffer any illness for which -according to the physician- you require medical evaluations at least every three months?”), and having health insurance (yes or no).

Consultation variables included in the analysis were: Having a scheduled appointment (evaluated with the yes/no question: “Did you have a scheduled appointment for the health service you received today?”), being accompanied (evaluated with the question “Have you come accompanied or alone?”), and reporting that the physician explained his/her health problem (evaluated with the yes/no question “Did the physician clearly explain your illness or health problem?”).

Facility variables included in the analysis were: ACF level (I, II, o III), health system (MOH-RG, Social Security, Armed Forces and Police, or private), and geographical region (coast, highlands, jungle, or the city of Lima).

### Ethics

This study is a secondary analysis of a public database.

### Data analysis

Data analysis was performed using STATA® version 14.0 (STATA Corporation, College Station, Texas, USA). We followed the ENSUSALUD-2015 sampling specifications, including stratification, expansion factor, and primary and secondary sampling units.

For descriptive analysis, absolute and relative frequencies were used for categorical variables, while mean with 95% confidence intervals (95% CI) were calculated for continuous variables. Mean waiting, consultation time, and patient satisfaction were calculated by each health care level, health system, and geographical region. Additionally, we used chi-2 test for bivariable analysis between patient satisfaction and health care level, health system, and geographical region.

Before undertaking the association analysis, we grouped the waiting time and consultation time variables, into 10 min intervals. Because we considered that the 10-min unit would be more helpful for the statistical and practical interpretation of the results, instead of the every-minute unit. In addition, since we considered an average waiting time of 90 min, 10 min intervals were considered appropriate. A similar approach, using minutes interval, were published elsewhere [12]. Then, to evaluate the relationship between independent variables (waiting time and consultation time, both evaluated by 10-min intervals) with patient satisfaction, crude and adjusted logistic regressions models for complex survey sampling were fit to estimate odds ratios (OR and aOR) and their 95% confidence intervals (CI). Logistic regression models were adjusted by age, sex, having finished secondary education, wealth, having a chronic disease, having health insurance, having a scheduled appointment, being accompanied, reporting that the physician explained his/her health problem, health care level, geographical region, health system, waiting time, and consultation time. To avoid the influence of outliers, we excluded observations with consultation time > 30 min in the consultation time/patient satisfaction associations (19 participants excluded, 0.14% of total population). Their responded consultation time ranged between 34 and 90 min. This was because we considered that a consultation time with more than 30 min is a very rare scenario, and including these observations in the regression analysis would bias the results. We didn’t exclude very short consultations times because we considered those were plausible in our context. Additionally, very short consultations are not likely to affect the regressions coefficients since they are close to the mean consultation time, unlike very long consultation times.

In addition, the associations were plotted using restricted cubic splines with five knots obtained by default, as previously suggested [27], in order to evaluate linearity and identify possibly cut-off points to difference subpopulations in which the association would be different.

Finally, we assessed the association between time and consultation time, using Pearson correlation test and, additionally we calculating the mean and 95% CI consultation time by each tercile of the waiting time.

## Results

### Population description

The ENSUSALUD-2015 included 13,670 participants from 181 ACFs. Response rate was 100% in satisfaction and waiting time, and 13,642 (99, 8%) participants responded consultation time. When including weights and design effect of the survey’s complex sampling, we found that 5335 (40.4%) were males, mean age was 42.8 years (95% CI: 41.3–44.3 years, range: 15–95 years), 9327 (72.0%) finished secondary education, 5473 (28.7%) were in the 1st or 2nd wealth quintile, and 9198 (73.7%) were satisfied with the care received (Table 1).

**Table 1:** General characteristics from Peruvian ambulatory care facilities’ users

Characteristics	Absolute frequency (percentages *)
Patients’ characteristics	
Age in years: mean (95% CI)	42.8 (41.3–44.3)
Females	8335 (59.6)
Finished secondary education	9327 (72.0)
1st or 2nd wealth quintile	5473 (28.7)
Have a chronic disease	5206 (51.2)
Have insurance	12,679 (92.2)
Consultation’ characteristics	
Scheduled appointment for consultation	8788 (66.3)
Came accompanied for consultation	4466 (34.8)
The physician explained his/her health problem	10,296 (77.7)
Facility’ characteristics	
Health care level	
Level I	2643 (29.1)
Level II	8626 (36.1)
Level III	2401 (34.8)
Health system	
MOH-RG	6410 (44.9)
Social Security	6081 (34.6)
Armed Forces and Police	524 (8.8)
Private Sector	655 (11.7)
Region	
Coast	4206 (23.7)
Highlands	5486 (18.5)
Jungle	2706 (6.6)
Lima city	1272 (51.3)
Patient satisfied	9198 (73.7)

*Weights and design effect of the survey’s complex sampling were included to calculate percentages

*CI* Confidence Interval, *MOH-RG* Ministry of Health and Regional Governments

Average waiting time was 104.2 min (95% CI: 91.0–117.4 min), and average consultation time was 12.1 min (95% CI: 11.4–12.7 min). Average waiting time was higher among users of MOH-RG ACFs (mean: 147.5, 95% CI: 129.3–165.8), and lower among users of armed forces and police ACFs (mean: 55.7, 95% CI: 37.5–73.9), and private ACFs (mean: 41.5, 95% CI: 34.7–48.3). With respect to the geographical region, users of ACFs of the city of Lima had a noticeably lower waiting time (mean: 94.6, 95% CI: 70.9–118.2) and higher average consultation time (mean: 13.0, 95% CI: 12.0–14.1) than other ACFs. Patient satisfaction was statistically greater in armed forces and police ACFs (90.9%), private ACFs (93.2%), and in users of ACFs of the city of Lima (81.6%) (chi-2 *p*-value to evaluate the association between patient satisfaction with health services and region *p* < 0,001) (Table 2).

**Table 2:** Waiting time and consultation time by variables categories

Subgroups	Mean (95% CI) *		Absolute Frequency (percentages *)
	Waiting time	Consultation time	Patient satisfaction
Overall population	104.2 (91.0–117.4)	12.1 (11.4–12.7)	9198 (73.7)
Health system**			
MOH-RG	147.5 (129.3–165.8)	12.3 (11.4–13.2)	4180 (72.0)
Social Security	81.5 (65.7–97.3)	11.3 (10.2–12.4)	3989 (64.9)
Armed Forces and Police	55.7 (37.5–73.9)	10.7 (8.6–12.8)	425 (90.9)
Private	41.5 (34.7–48.3)	14.4 (12.1–16.8)	604 (93.2)
Health care level			
Level I	110.9 (83.2–138.6)	10.6 (9.3–12.0)	1824 (73.0)
Level II	97.6 (79.9–115.2)	11.9 (10.8–13.0)	5770 (72.3)
Level III	105.5 (78.6–132.4)	13.4 (12.6–14.2)	1604 (75.8)
Region**			
Coast	116.4 (98.3–134.5)	11.2 (9.9–12.4)	2618 (64.1)
Highlands	109.2 (85.3–133.0)	11.1 (10.5–11.8)	2006 (74.4)
Jungle	121.6 (103.3–139.9)	10.6 (10.1–11.0)	3536 (63.9)
Lima city	94.6 (70.9–118.2)	13.0 (12.0–14.1)	1038 (81.6)

*Weights and design effect of the survey’s complex sampling were included to calculate percentages. **p < 0,001 for chi-2 test for evaluation between patient satisfaction and variables. *CI* Confidence Interval, *MOH-RG* Ministry of Health and Regional Governments

### Association of waiting time and consultation time with patient satisfaction

**Fig. 1:**
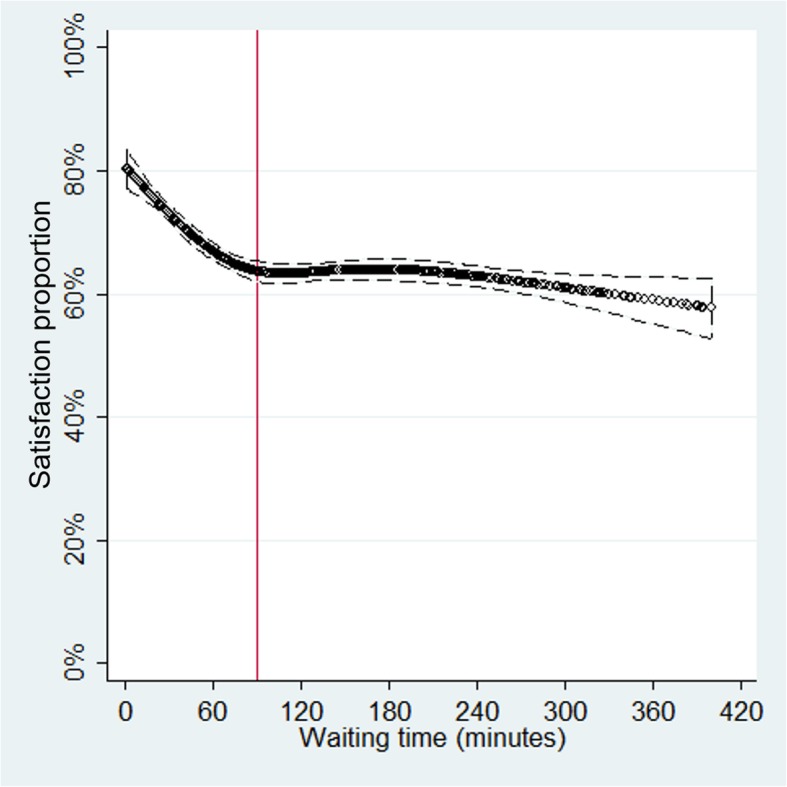
Association between waiting time and proportion of patient satisfaction, using restricted cubic splines (red line at 90 min)

Waiting time (for every 10 min) had a small inverse associated with patient satisfaction (aOR: 0.98, 95% CI: 0.97–0.99). However, the plot showed that this association was stronger among those who reported waiting time ≤ 90 min, but was almost absent for those who reported waiting time > 90 min. This was corroborated with the logistic regressions performed in both subpopulations: those who reported waiting time ≤ 90 min and > 90 min (Table 3 and Fig. 1).

**Table 3:** Multivariate analysis of waiting time and consultation time with patient satisfaction

	Crude	Adjusted
	OR (95% CI)	aOR (95% CI)*
Exposure: Waiting time (for every 10 min)		
Overall population	**0.97 (0.96–0.98)**	**0.98 (0.97–0.99)**
Subgroup: Waiting time ≤ 90 min	**0.89 (0.84–0.94)**	**0.92 (0.89–0.96)**
Subgroup: Waiting time > 90 min	0.99 (0.98–1.00)	0.99 (0.97–1.00)
Exposure: Consultation time (for every 10 min)		
Overall population	**1.92 (1.41–2.61)**	**1.59 (1.26–2.01)**
Subgroup: Consultation time ≤ 15 min	**2.70 (1.83–3.98)**	**2.31 (1.66–3.21)**
Subgroup: Consultation time > 15 min	0.90 (0.42–1.93)	0.89 (0.34–2.29)

*Adjusted by: age, sex, having finished secondary education, wealth, having a chronic disease, having health insurance, having a scheduled appointment, being accompanied, reporting that the physician explained about his/her health problem, health care level, geographical region, health system, waiting time, and consultation time. **Bold results**: Statistical significance (*p* < 0.05)

**Fig. 2:**
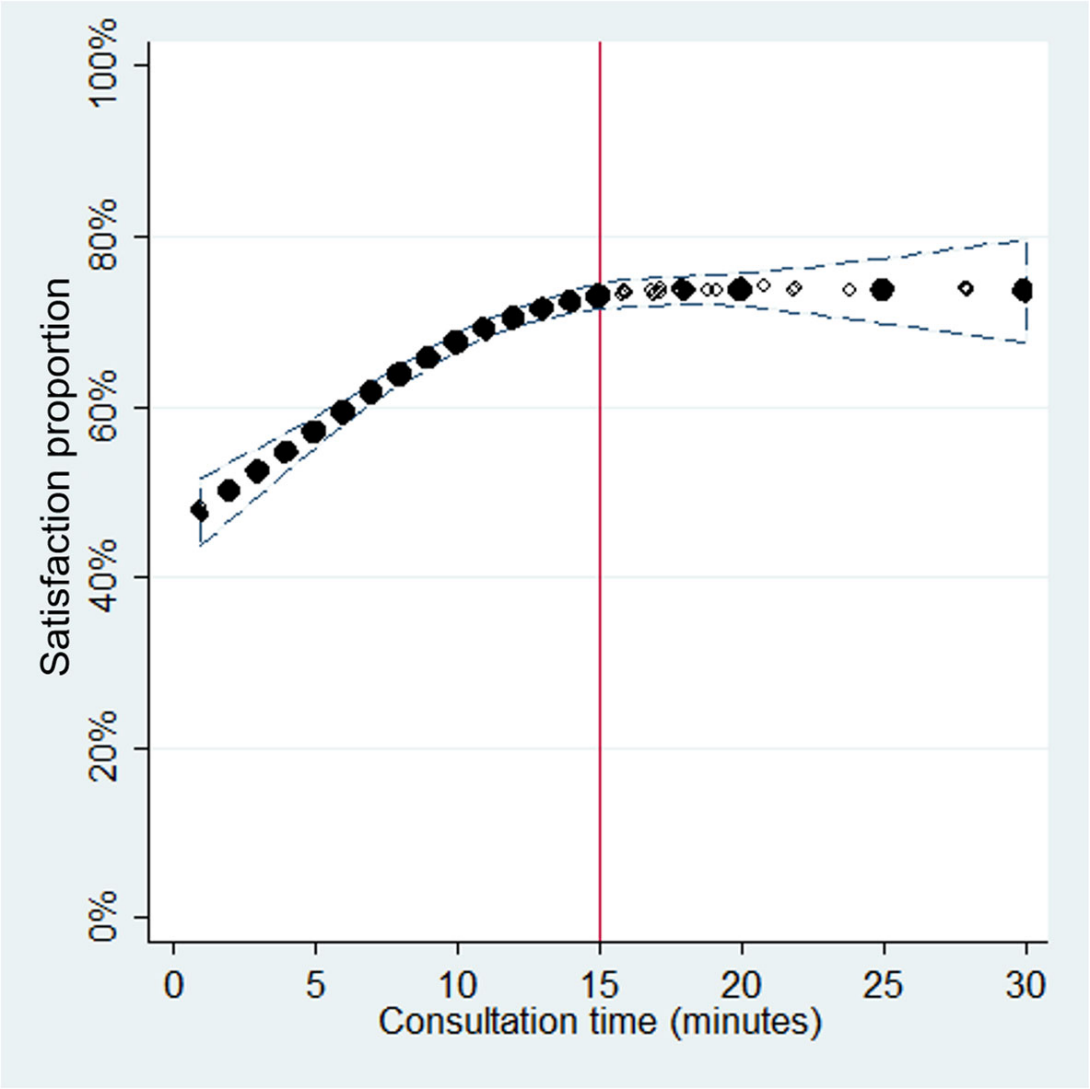
Association between consultation time and proportion of patient satisfaction, using restricted cubic splines (red line at 15 min)

In the consultation time and patient satisfaction associations, 19 observations with consultation time > 30 min were excluded of the analysis, in order to avoid the influence of outliers. Consultation time (for every 10 min) was directly associated with patient satisfaction (aOR: 1.59, 95% CI: 1.26–2.01). However, the plot showed that this association was stronger among those who reported consultation time ≤ 15 min, but was almost absent for those who reported consultation time > 15 min. This was corroborated with the logistic regressions performed in both subpopulations: those who reported consultation time ≤ 15 min and > 15 min (Table 3 and Fig. 2).

Finally, the mean and 95% CI consultation time in the first, second, and third tercile of the waiting time were: 12.4 (95% CI: 11.4 to 13.4), 12.2 (95% CI: 11.5 to 12.8), and 11.6 (95% CI: 10.7 to 12.5) minutes, respectively. The correlation between time and consultation time was not statistically significant (*p* = 0.0819).

## Discussion

### Main results

In Peruvian ACFs, average waiting time was 104.2 min and average consultation time was 12.1 min. Waiting time was slightly inversely associated with patient satisfaction, and this association was statistically significant only among those who reported waiting time ≤ 90 min. Likewise, Consultation time was directly associated with patient satisfaction, and this association was more notable among individuals who reported consultation time ≤ 15 min.

### Association between waiting time and patient satisfaction

Average waiting time in this study was 104.2 min, which is longer than average waiting time found in high-income or upper-middle countries such as Germany (13.7 min) [16], USA (21 min) [14], and Mexico (30 min) [28]. But lower to average waiting time reported in middle and low-income countries like Uganda (2–4 h) [29], Malaysia (143.4 min) [26], and Nigeria (131.1 min) [30].

The present study reports that waiting time in Peruvian ACFs is inversely associated with patient satisfaction, coinciding with a previous study developed in an endocrinology outpatient department where the waiting time was negatively associated with patient satisfaction of the care they received [31]. This association showed a plateau among those who reported waiting time > 90 min. This report and suggested cut-off point are new in the Latin-American region context. A previous study in the USA also reported a plateau among those with waiting time > ~ 25 min, but only in patients who had more than 10 min of consultation time. However, other similar studies undertaken in the USA have reported linear trends without plateaus [10, 14]. This was likely due to the waiting time range found in those studies being smaller than ours: waiting time ranged between 1 and 60 min [14], and between 1 and 15 min [10].

This plateau might indicate that users who wait more than 90 min take into account the quality of the ACF or the consulted physician’s long waiting time and therefore has planned to invest a long time waiting, or maybe there is a sub-population who expect to wait for care. However, an alternative explanation is that most individuals go to the ACF in the morning, as reported in the final report of ENSUSALUD-2015 [32]. So, they expect to return to their work or their home in a short period of time, and when made to wait > 90 min, permission to be late at work have already been overwhelmed, while domestic activities (such as cooking or bringing children to school) are no longer required; so from this point longer times have smaller consequences. This hypothesis can be feasible because people give their own value of time depending on the context: If they don’t have anything to lose, their value of time is lower [33].

Therefore, it is possible that interventions which aim to reduce waiting time within the range of the first 90 min have an impact in patient satisfaction, in accordance with previous reports [15, 34–36]. While reducing waiting time between ranges higher than 90 min may have little or no impact on satisfaction. However, we did not found interventions that have evaluated reductions in waiting time beyond the range of the first 90 min. In addition, it would be useful to qualitatively assess why participants do not appear to have a significant change on in their satisfaction when waiting time exceeds 90 min.

### Association between consultation time and patient satisfaction

Average consultation time was 12.1 min, similar to the average consultation time reported in Malaysia (12.8 min) [26], and longer than those reported in Bangladesh (2.3 min) [8] and United Kingdom (10.4 min) [17]. Despite this, the Peruvian Ministry of Health recommends that consultation time should last between 15 to 30 min [37], and The Association of Chiefs and Leaders in General Internal Medicine of USA, recommends that it should last ≥30 min to provide high-quality care [38].

Consultation time and patient satisfaction were directly associated, similar to results found in studies performed in Sweden [15] and USA. Conversely, a study performed in England [17] and a study performed also in the USA [39] found no association between consultation time and patient satisfaction, possibly because these last studies were performed in contexts that differed from ours (only in primary care health services, and only in ambulatory care in hand surgery, respectively). Particularly, in the last study where the consultation time range where narrower between 4 and 16 min [39] than the first mentioned study in USA between 6 and 72 min.

This association showed a plateau among those with > 15 min of consultation time, which suggests that increasing the consultation time over this cut-off point may have little effect on patient satisfaction. This may be because patients are not willing to invest more time than they deem necessary in the consultation, or maybe these patients report very high levels of satisfaction after that time, which could not be measured with the 5-points Likert scale used. This results could explain why average consultation time appears to be positively associated with some, but not all, elements of patient satisfaction [40]. Therefore, longer consultation times could have no effect on satisfaction which should be expanded by further interventional or qualitative studies. This result is new since we have not found other studies that had have plotted this association.

### Limits and strengths

It is necessary to emphasize some limitations from the present study: 1) Patient satisfaction is a complex construct [41], so it is possible that the question used to collect it is insufficient to address its complexity. However, some previous studies have also collected this variable using single questions [17, 42]. 2) Waiting and consultation times have been measured by self-reporting, which may present memory bias comparing with reliable instruments such as direct observation or video or audio recordings [17, 43], and there is the likelihood that dissatisfied patients estimate their waiting time longer than satisfied patients. However, the memory bias should be minimized since these times were asked immediately following the patient’s consultation with the physician, and also previous reports used this methodology [28]. 3) We couldn’t evaluate other variables, such as receptionist’s attitude, suitability of waiting area, privacy during the consultation, quality of consultation time, which could affect the evaluated relationships. However, this is an inherent limitation in all secondary analysis studies.

Despite those limitations, this is one of the first studies in a middle low-income country that delves into the linear association of waiting time and consultation time with patient satisfaction. In addition, the sampling strategy and the size sample of the analyzed database allows a national-level inferential analysis, so our results could be of interest for the management of ACFs in Peru and in other countries with similar contexts. These new results suggest that interventions with the aim to increase patient satisfaction should be oriented toward a decrease in waiting time < 90 min and increase consultation time to a maximum of 15 min, in a low and middle-low country context. However, these proposed cut-off points must have been taking into account in a general context of ACFs, and additionally, we have to consider other variables as the context and culture of the service and the population. Finally, we must consider patient satisfaction as a complex outcome, which is also influenced by other variables [44].

## Conclusion

In Peruvian ACFs, both waiting time and consultation time showed a non-linear association with overall patient satisfaction, which was stronger in the first 90 min of waiting time and in the first 15 min of consultation time, respectively. This should be taken into consideration when designing interventions to improve waiting times and consultation times in ACFs from Peru or from similar contexts.

## Data Availability

The database and details of the survey are public on the INEI webpage (http://iinei.inei.gob.pe/microdatos/).

## Abbreviations

ACFs: Ambulatory care facilities
ENSUSALUD-2015, in Spanish: National Survey on User Satisfaction of Health Services 2015
EsSalud: Social Security
INEI, in Spanish: National Institute of Statistics and Informatics
MOH-RG: Ministry of Health and Regional Governments
OR: Odds Ratio
SUSALUD, in Spanish: National Superintendence of Health
